# An Axiomatic Approach to Reversible Computation

**DOI:** 10.1007/978-3-030-45231-5_23

**Published:** 2020-04-17

**Authors:** Ivan Lanese, Iain Phillips, Irek Ulidowski

**Affiliations:** 8 ENS/CNRS, Université Paris-Saclay, Cachan, France; 9 University of Duisburg-Essen, Duisburg, Germany; 10 Focus Team, University of Bologna/INRIA, Bologna, Italy; 11 Imperial College London, London, England; 12 University of Leicester, Leicester, England

**Keywords:** Reversible Computation, Labelled Transition System with Independence, Causal Safety, Causal Liveness

## Abstract

Undoing computations of a concurrent system is beneficial in many situations, e.g., in reversible debugging of multi-threaded programs and in recovery from errors due to optimistic execution in parallel discrete event simulation. A number of approaches have been proposed for how to reverse formal models of concurrent computation including process calculi such as CCS, languages like Erlang, prime event structures and occurrence nets. However it has not been settled what properties a reversible system should enjoy, nor how the various properties that have been suggested, such as the parabolic lemma and the causal-consistency property, are related. We contribute to a solution to these issues by using a generic labelled transition system equipped with a relation capturing whether transitions are independent to explore the implications between these properties. In particular, we show how they are derivable from a set of axioms. Our intention is that when establishing properties of some formalism it will be easier to verify the axioms rather than proving properties such as the parabolic lemma directly. We also introduce two new notions related to causal consistent reversibility, namely causal safety and causal liveness, and show that they are derivable from our axioms.

## 1 Introduction

Reversible computing studies computations which can proceed both in the standard, forward direction, and backward, going back to past states. Reversible computation has attracted interest due to its applications in areas as different as low-power computing [15], simulation [4], robotics [21], biological modelling [31] and debugging [23].

* This work has been partially supported by COST Action IC1405 on Reversible Computation - Extending Horizons of Computing. The first author has also been partially supported by French ANR project DCore ANR-18-CE25-0007 and by INdAM as a member of GNCS (Gruppo Nazionale per il Calcolo Scientifico).

There is widespread agreement in the literature about what properties characterise reversible computation in the sequential setting. Thus in reversible finite state automata [32], reversible cellular automata [13], reversible Turing machines [2] and reversible programming languages such as Janus [35] the main point is that the mapping from inputs to outputs is injective, and the reverse computation is deterministic.

Matters are less clear when it comes to reversible computation in the concurrent setting. Indeed, various reversible concurrent models have been studied, most notably in the areas of process calculi [6,29,18], event structures [34], Petri nets [1,25] and programming languages such as Erlang [20].

A main result of this line of research is that the notion of reversibility most suited for concurrent systems is *causal-consistent reversibility* (other notions are also used, e.g., to model biological systems [31]). According to an informal account of causal-consistent reversibility, any action can be undone provided that its consequences, if any, are undone beforehand. Following [6] this account is formalised using the notion of causal equivalent traces: two traces are causal equivalent if and only if they only differ for swapping independent actions, and inserting or removing pairs of an action and its reverse. According to [6, Section 3]

> Backtracking an event is possible when and only when a causally equivalent trace would have brought this event as the last one which is then formalised as the so called causal consistency (CC) [6, Theorem 1], stating that coinitial computations are causal equivalent if and only if they are cofinal. Our new proof of CC (Proposition 3.6) shows that it holds in essentially any reversible formalism satisfying the Loop Lemma and the Parabolic Lemma, and we believe that CC is insufficient on its own to capture the informal notion.

A formalisation closer to the informal statement above is provided in [20, Corollary 22], stating that a forward transition $t$ can be undone after a derivation iff all its consequences, if any, are undone beforehand. We are not aware of other discussions trying to formalise such a notion, except for [30], in the setting of reversible event structures. In [30], a reversible event structure is *cause-respecting* if an event cannot be reversed until all events it has caused have also been reversed; it is *causal* if it is cause-respecting and a reversible event can be reversed if all events it has caused have been reversed [30, Definition 3.34].

We provide (Section 4) a novel definition of the idea above, composed by:

**Causal Safety (CS):** an action cannot be reversed until any actions caused by it have been reversed;

**Causal Liveness (CL):** we should allow actions to reverse in any order compatible with CS, not necessarily the exact inverse of the forward order.

We shall see that CC does not capture the same property as CS+CL (Examples 4.15, 4.37), and that there are slightly different versions of CS and CL, which can all be proved under a small set of reasonable assumptions.

The main aim of this paper is to take an abstract model, namely labelled transition systems with independence equipped with reverse transitions (Section 2), and to show that the properties above (as well as others) can be derived

| Acronym | Name | Defined in | Proved in | using |
|---|---|---|---|---|
| SP | Square Property | Def. 3.1 | Axiom | - |
| BTI | Backward Transitions are Independent | Def. 3.1 | Axiom | - |
| WF | Well-Founded | Def. 3.1 | Axiom | - |
| CPI | Coinitial Propagation of Independence | Def. 4.2 | Axiom | - |
| IRE | Independence Respects Events | Def. 4.12 | Axiom | - |
| CIRE | Coinitial Independence Respects Events | Def. 4.29 | Axiom | implied by IRE |
| IEC | Independence of Events is Coinitial | Def. 4.16 | Axiom | - |
| PL | Parabolic Lemma | Def. 3.3 | Prop. 3.4 | BTI, SP |
| CC | Causal Consistency | Def. 3.5 | Prop. 3.6 | WF, PL |
| UT | Unique Transition | Def. 3.7 | Cor. 3.8 | CC |
| ID | Independence of Diamonds | Def. 4.6 | Prop. 4.7 | BTI, CPI |
| RPI | Reversing Preserves Independence | Def. 4.17 | Prop. 4.18 | SP, CPI, IRE, IEC |
| CS | Causal Safety | Def. 4.11 | Thm. 4.13 | SP, BTI, WF, CPI, IRE |
| CL | Causal Liveness | Def. 4.11 | Thm. 4.14 | SP, BTI, WF, CPI, IRE |
| $\text{CS}_<$ | ordered Causal Safety | Def. 4.24 | Prop. 4.39 | SP, BTI, WF, CPI, NRE |
| $\text{CL}_<$ | ordered Causal Liveness | Def. 4.24 | Prop. 4.39 | SP, BTI, WF, CPI, CIRE |
| $\text{CS}_{\text{ci}}$ | coinitial Causal Safety | Def. 4.27 | Thm. 4.28 | SP, BTI, WF, CPI |
| $\text{CL}_{\text{ci}}$ | coinitial Causal Liveness | Def. 4.27 | Thm. 4.30 | SP, BTI, WF, CPI, CIRE |
| NRE | No Repeated Events | Def. 4.35 | Prop. 4.42 | SP, BTI, WF, CPI, CIRE |
| RED | Reverse Event Determinism | Def. 4.40 | Prop. 4.41 | SP, BTI, WF, CPI, NRE |

**Table 1.** Axioms and properties for causal reversibility.

from a small set of simple axioms (Sections 3, 4, 5). This is in sharp contrast with the large part of works in the literature, which consider specific frameworks such as CCS [6], CCS with broadcast [26], CCB [14], $\pi$-calculus [5], higher-order $\pi$ [18], Klaim [11], Petri nets [25], $\mu$Oz [22] and Erlang [20], and all give similar but formally unrelated proofs of the same main results. Such proofs will become instances of our general results. More precisely, our axioms will:

- exclude behaviours which are not compatible with causal-consistent reversibility (as we will discuss shortly);
- allow us to derive the main properties of reversible calculi which have been studied in the literature, such as CC (Proposition 3.6);
- hold for a number of reversible calculi which have been proposed, such as RCCS [6] and reversible Erlang [20] (Section 6).

Thus, when defining a new reversible formalism, one just has to check whether the axioms hold, and get for free the proofs of the most relevant properties. Notably, the axioms are normally easier to prove than the properties, hence the assessment of a reversible calculus gets much simpler.

As a reference, Table 1 lists the axioms and properties used in this paper.

In order to understand which kinds of behaviours are incompatible with a causal-consistent reversible setting, consider the following LTSs in CCS:

$a.\mathbf{0} \xrightarrow{a} \mathbf{0}$**,** $b.\mathbf{0} \xrightarrow{b} \mathbf{0}$**:** from state $\mathbf{0}$ one does not know whether to go back to $a.\mathbf{0}$ or to $b.\mathbf{0}$;

$a.\mathbf{0} + b.\mathbf{0} \xrightarrow{a} \mathbf{0}$**,** $a.\mathbf{0} + b.\mathbf{0} \xrightarrow{b} \mathbf{0}$**:** as above, but starting from the same process, hence showing that it is not enough to remember the initial configuration;

$P \xrightarrow{a} P$ **where** $P = a.P$**:** one can go back forever, against the idea that a state models a process reachable after a finite computation.

We remark that all such behaviours are perfectly reasonable in CCS, and they are dealt with in the reversible setting by adding history information about past actions. For example, in the first case one could remember the initial state, in the second case both the initial state and the action taken, and in the last case the number of iterations that have been performed.

Due to space constraints, some proofs and additional results can only be found in the companion technical report [16].

## 2 Labelled Transition Systems with Independence

We want to study reversibility in a setting as general as possible. Thus, we base on the core of the notion of *labelled transition system with independence* (LTSI) [33, Definition 3.7]. However, while [33] requires a number of axioms on LTSI, we take the basic definition and explore what can be done by adding or not adding various axioms. Also, we extend LTSI with reverse transitions, since we study reversible systems. We define first labelled transition systems (LTSs).

We consider the LTS of the entire set of processes in a calculus, rather than the transition graph of a particular process and its derivatives, hence we do not fix an initial state.

**Definition 2.1.** *A* labelled transition system (LTS) *is a structure* $(\mathsf{Proc}, \mathsf{Lab}, \rightharpoonup)$*, where* $\mathsf{Proc}$ *is the set of states (or processes),* $\mathsf{Lab}$ *is the set of action labels and* $\rightharpoonup \subseteq \mathsf{Proc} \times \mathsf{Lab} \times \mathsf{Proc}$ *is a* transition relation*.*

We let $P, Q, \ldots$ range over processes, $a, b, c, \ldots$ range over labels, and $t, u, v, \ldots$ range over transitions. We can write $t : P \xrightarrow{a} Q$ to denote that $t = (P, a, Q)$. We call $a$-transition a transition with label $a$.

**Definition 2.2 (LTS with independence).** *We say that* $(\mathsf{Proc}, \mathsf{Lab}, \rightarrow, \iota)$ *is an* LTS with independence *(LTSI) if* $(\mathsf{Proc}, \mathsf{Lab}, \rightarrow)$ *is an LTS and* $\iota$ *is an irreflexive symmetric binary relation on transitions.*

In many cases (see Section 6), the notion of independence coincides with the notion of concurrency. However, this is not always the case. Indeed, concurrency implies that transitions are independent since they happen in different processses, but transitions taken by the same process can be independent as well. Think, for instance, of a reactive process that may react in any order to two events arriving at the same time, and the final result does not depend on the order of reactions.

We shall assume that all transitions are reversible, so that the Loop Lemma [6, Lemma 6] holds. This does not hold in models of reversibility with control mechanisms such as irreversible actions [6,7] or a rollback operator [17]. Nevertheless, when showing properties of models with controlled reversibility it has proved sensible to first consider the underlying models where all transitions are reversible, and then study how control mechanisms change the picture [11,20]. The present work helps with the first step.

**Definition 2.3.** *Given* $(\mathsf{Proc}, \mathsf{Lab}, \rightharpoonup)$, *let the* reverse LTS *be* $(\mathsf{Proc}, \mathsf{Lab}, \rightsquigarrow)$, *where* $P \stackrel{a}{\rightsquigarrow} Q$ *iff* $Q \stackrel{a}{\rightharpoonup} P$. *It is convenient to combine the two LTSs (forward and reverse): let the reverse labels be* $\underline{\mathsf{Lab}} = \{\underline{a} : a \in \mathsf{Lab}\}$, *and define the combined LTS to be* $\rightarrow \subseteq \mathsf{Proc} \times (\mathsf{Lab} \cup \underline{\mathsf{Lab}}) \times \mathsf{Proc}$ *by* $P \stackrel{a}{\rightarrow} Q$ *iff* $P \stackrel{a}{\rightharpoonup} Q$ *and* $P \stackrel{\underline{a}}{\rightarrow} Q$ *iff* $P \stackrel{a}{\rightsquigarrow} Q$.

We stipulate that the union $\mathsf{Lab} \cup \underline{\mathsf{Lab}}$ is disjoint. We let $\alpha, \ldots$ range over $\mathsf{Lab} \cup \underline{\mathsf{Lab}}$. For $\alpha \in \mathsf{Lab} \cup \underline{\mathsf{Lab}}$, the *underlying* action label $\mathsf{und}(\alpha)$ is defined as $\mathsf{und}(a) = a$ and $\mathsf{und}(\underline{a}) = a$. Let $\underline{\underline{a}} = a$ for $a \in \mathsf{Lab}$. Given $t : P \stackrel{\alpha}{\rightarrow} Q$, let $\underline{t} : Q \stackrel{\underline{\alpha}}{\rightarrow} P$ be the transition which reverses $t$.

We let $\rho, \sigma, \ldots$ range over finite sequences $\alpha_1 \ldots \alpha_n$, with $\varepsilon_P$ representing the empty sequence starting and ending at $P$. We shall write $\varepsilon$ when $P$ is understood. Given an LTS, a *path* is a sequence of forward or reverse transitions of the form $P_0 \stackrel{\alpha_1}{\rightarrow} P_1 \cdots \stackrel{\alpha_n}{\rightarrow} P_n$. We let $r, s, \ldots$ range over paths. We may write $r : P \stackrel{\rho}{\rightarrow}_* Q$ where the intermediate states are understood. On occasion we may refer to a path simply by its sequence of labels $\rho$. Given a path $r : P \stackrel{\rho}{\rightarrow}_* Q$, the inverse path is $\underline{r} : Q \stackrel{\underline{\rho}}{\rightarrow}_* P$ where $\underline{\varepsilon} = \varepsilon$ and $\underline{\alpha\rho} = \underline{\rho}\,\underline{\alpha}$. The length of a path $r$ (notated $|r|$) is the number of transitions in the path. Paths $r : P \stackrel{\rho}{\rightarrow}_* Q$ and $R \stackrel{\sigma}{\rightarrow}_* S$ are *coinitial* if $P = R$ and *cofinal* if $Q = S$. We say that a path is *forward-only* if it contains no reverse transitions.

Let $(\mathsf{Proc}, \mathsf{Lab}, \rightarrow)$ be an LTS. The irreversible processes in $(\mathsf{Proc}, \mathsf{Lab}, \rightarrow)$ are $\mathsf{Irr} = \{P \in \mathsf{Proc} : P \not\rightsquigarrow\}$. A *rooted path* is a path $r : P \stackrel{\rho}{\rightarrow}_* Q$ such that $P \in \mathsf{Irr}$.

In the following we will consider LTSIs obtained by adding a notion of independence to combined LTSs as above. We will call the result a *combined LTSI*.

## 3 Basic Properties

In this section we show that most of the properties in the reversibility literature (see, e.g., [6,29,18,20]), in particular the parabolic lemma and causal consistency, can be proved under minimal assumptions on the combined LTSI under analysis.

We formalise the minimal assumptions using three axioms, described below.

**Definition 3.1 (Basic axioms).** *Let* $\mathcal{L} = (\mathsf{Proc}, \mathsf{Lab}, \rightarrow, \iota)$ *be a combined LTSI. We say* $\mathcal{L}$ *satisfies:*

**Square Property (SP)** *if whenever* $t : P \stackrel{\alpha}{\rightarrow} Q$, $u : P \stackrel{\beta}{\rightarrow} R$ *with* $t \;\iota\; u$ *then there are cofinal transitions* $u' : Q \stackrel{\beta}{\rightarrow} S$ *and* $t' : R \stackrel{\alpha}{\rightarrow} S$;

**Backward Transitions are Independent (BTI)** *if whenever* $t : P \stackrel{a}{\rightsquigarrow} Q$ *and* $t' : P \stackrel{b}{\rightsquigarrow} Q'$ *and* $t \neq t'$ *then* $t \;\iota\; t'$;

**Well-Foundedness (WF)** *if there is no infinite reverse computation, i.e. we do not have* $P_i$ *(not necessarily distinct) such that* $P_{i+1} \stackrel{a_i}{\rightsquigarrow} P_i$ *for all* $i = 0, 1, \ldots$.

WF can alternatively be formulated using backward transitions, but the current formulation makes sense also in non-reversible calculi (e.g., CCS), which can be used as a comparison. Let us discuss the intuition behind these axioms. SP takes its name from the Square Lemma, where it is proved for concrete calculi and languages in [6,18,20], and captures the idea that independent transitions can be executed in any order, that is they form commuting diamonds. SP can be seen as a sanity check on the chosen notion of independence. BTI generalises the key notion of backward determinism used in sequential reversibility (see, e.g., [32] for finite state automata and [35] for the imperative language Janus) to a concurrent setting. Backward determinism can be spelled as "two coinitial backward transitions do coincide". This can be generalised to "two coinitial backward transitions are independent". Finally, WF means that we consider systems which have a finite past. That is, we consider systems starting from some initial state and then moving forward and back.

Axioms SP and BTI are related to properties which are part of the definition of (occurrence) transition systems with independence in [33, Definitions 3.7, 4.1]. WF was used as an axiom in [28].

Using the minimal assumptions above we can prove relevant results from the literature. We first define causal equivalence, equating computations differing only for swaps of independent transitions and simplification of a transition with its reverse.

**Definition 3.2 (cf. [6]).** *Let* $(\mathsf{Proc}, \mathsf{Lab}, \rightarrow, \iota)$ *be an LTSI satisfying SP. Let* $\approx$ *be the smallest equivalence relation on paths closed under composition and satisfying:*

1. *if* $t : P \xrightarrow{\alpha} Q$, $u : P \xrightarrow{\beta} R$ *are independent, and* $u' : Q \xrightarrow{\beta} S$, $t' : R \xrightarrow{\alpha} S$ *(which exist by SP) then* $tu' \approx ut'$*;*
2. $t\underline{t} \approx \varepsilon$ *and* $\underline{t}t \approx \varepsilon$*.*

We first consider the Parabolic Lemma ([6, Lemma 10]), which states that each path is causal equivalent to a backward path followed by a forward path.

**Definition 3.3. Parabolic Lemma (PL)***: for any path* $r$ *there are forward-only paths* $s, s'$ *such that* $r \approx \underline{s}s'$ *and* $|s| + |s'| \leq |r|$*.*

**Proposition 3.4.** *Suppose an LTSI satisfies BTI and SP. Then PL holds.*

The proof of Proposition 3.4 (available in [16]) is very similar to that of [6, Lemma 10] except that in the latter BTI is shown directly as part of the proof.

A corollary of PL is that if a process is reachable from an irreversible process, then it is also forwards reachable from it. In other words, making a system reversible does not introduce new reachable states but only allows one to explore differently forwards reachable states. This is relevant in reversible debugging of concurrent systems [10,20], where one wants to find bugs that actually occur in forward-only computations. See the companion technical report [16, Corollary A.1]. We now move to causal consistency [6, Theorem 1].

**Definition 3.5. Causal Consistency (CC)**: *if $r$ and $s$ are coinitial and cofinal then $r \approx s$.*

Essentially, causal consistency states that history information allows one to distinguish computations which are not causal equivalent, indeed, if two computations are cofinal, that is they reach the same final state (which includes the stored history information) then they need to be causal equivalent.

Causal consistency frequently includes the other direction, namely that coinitial causal equivalent computations are cofinal, meaning that there is no way to distinguish causal equivalent computations. This second direction follows easily from the definition of causal equivalence.

Notably, our proof of CC below is very much shorter than existing proofs.

**Proposition 3.6.** *Suppose an LTSI satisfies WF and PL. Then CC holds.*

*Proof.* Let $r : P \xrightarrow{\rho}_* Q$ and $r' : P \xrightarrow{\rho'}_* Q$. Using WF, let $I, s$ be such that $s : I \xrightarrow{\sigma}_* P$, $I \in \mathsf{Irr}$. Now $sr\underline{sr'}$ is a path from $I$ to $I$, and so by PL there are $r_1, r_2$ forward-only such that $\underline{r_1}r_2 \approx sr\underline{sr'}$. But $I \in \mathsf{Irr}$ and so $r_1 = \varepsilon$ and $r_2 = \varepsilon$. Thus $\varepsilon \approx sr\underline{sr'}$, so that $sr \approx sr'$ and $r \approx r'$ as required. □

Causal consistency implies the unique transition property.

**Definition 3.7.** *An LTSI* $(\mathsf{Proc}, \mathsf{Lab}, \rightarrow, \iota)$ *satisfies **Unique Transition (UT)** if $P \xrightarrow{a} Q$ and $P \xrightarrow{b} Q$ imply $a = b$.*

**Corollary 3.8.** *If an LTSI satisfies CC then it satisfies UT.*

UT was shown in the forward-only setting of occurrence TSIs in [33, Corollary 4.4]; it was taken as an axiom in [28].

*Example 3.9 (PL alone does not imply WF or CC).* Consider the LTSI with states $P_i$ for $i = 0, 1, \ldots$ and transitions $t_i : P_{i+1} \xrightarrow{a} P_i$, $u_i : P_{i+1} \xrightarrow{b} P_i$ with $a \neq b$ and $t_i \; \iota \; u_i$. BTI and SP hold. Hence PL holds by Proposition 3.4. However clearly WF fails. Also $t_i$ and $u_i$ are coinitial and cofinal, and $a \neq b$, so that UT fails, and hence CC fails using Corollary 3.8. Note that the $ab$ diamonds here have the same side states so are degenerate (cf. Lemma 4.4).

## 4 Causal Safety and Causal Liveness

In the literature, causal consistent reversibility is frequently informally described by saying that "a transition can be undone if and only if each of its consequences, if any, has been undone". In this section we study this property, where the two implications will be referred to as causal safety and causal liveness. We provide three different versions of such properties, based on independence of transitions (Section 4.2), ordering of events (Section 4.3), and independence of events (Section 4.4), and study their relationships. In order to define such properties we need the concept of event.

### 4.1 Events

**Definition 4.1 (Event, general definition).** *Let* $(\mathsf{Proc}, \mathsf{Lab}, \to, \iota)$ *be an LTSI. Let* $\sim$ *be the smallest equivalence relation satisfying: if* $t : P \xrightarrow{\alpha} Q$, $u : P \xrightarrow{\beta} R$, $u' : Q \xrightarrow{\beta} S$, $t' : R \xrightarrow{\alpha} S$, *and* $t \mathrel{\iota} u$, $\underline{u} \mathrel{\iota} t'$, $\underline{t'} \mathrel{\iota} \underline{u'}$, $u' \mathrel{\iota} \underline{t}$, *and*

- $Q \neq R$ *if* $\alpha$ *and* $\beta$ *are both forwards or both backwards;*
- $P \neq S$ *otherwise;*

*then* $t \sim t'$. *The equivalence classes of forward transitions, written* $[P, a, Q]$, *are the* events. *The equivalence classes of reverse transitions, written* $[P, \underline{a}, Q]$, *are the* reverse events. *Define a labelling function* $\ell$ *from* $\to / \sim$ *to* $\mathsf{Lab}$ *by setting* $\ell([P, \alpha, Q]) = \alpha$.

Events are introduced as a derived notion in an LTS with independence in [33], in the context of forward-only computation. We have changed their definition by using coinitial independence at all corners of the diamond, yielding rotational symmetry. This reflects our view that forward and backward transitions have equal status.

Our definition can be simplified if the LTSI, and independence in particular, are well-behaved. Thus, we now add a further axiom related to independence.

**Definition 4.2 (Coinitial Propagation of Independence (CPI)).** *If* $t : P \xrightarrow{\alpha} Q$, $u : P \xrightarrow{\beta} R$, $u' : Q \xrightarrow{\beta} S$ *and* $t' : R \xrightarrow{\alpha} S$ *with* $t \mathrel{\iota} u$, *then* $u' \mathrel{\iota} \underline{t}$.

CPI states that independence is a property of commuting diamonds more than of their specific pairs of edges. Indeed, it allows independence to propagate around a commuting diamond.

**Definition 4.3.** *If a combined LTSI satisfies axioms SP, BTI, WF and CPI, we say that it is* pre-reversible.

The name 'pre-reversible' indicates that we expect to require further axioms, but the present four are enough to ensure that LTSIs are well-behaved, with events compatible with causal equivalence. Pre-reversible axioms are separated from further axioms by a dashed line in Table 1.

The following non-degeneracy property was shown for occurrence transition systems with independence in [33, page 312], which have forward transitions only. We have to cope with backwards as well as forward transitions.

**Lemma 4.4.** *Suppose that an LTSI is pre-reversible. If we have a diamond* $t : P \xrightarrow{\alpha} Q$, $u : P \xrightarrow{\beta} R$ *with* $t \mathrel{\iota} u$ *together with cofinal transitions* $u' : Q \xrightarrow{\beta} S$ *and* $t' : R \xrightarrow{\alpha} S$, *then the diamond is* non-degenerate, *meaning that* $P, Q, R, S$ *are distinct states.*

If an LTSI is pre-reversible then by Lemma 4.4 and the use of CPI we can simplify the statement of Definition 4.1 to:

**Definition 4.5 (Event, simplified definition).** *Let* $(\mathsf{Proc}, \mathsf{Lab}, \rightarrow, \iota)$ *be a pre-reversible LTSI. Let* $\sim$ *be the smallest equivalence relation satisfying: if* $t : P \xrightarrow{\alpha} Q$, $u : P \xrightarrow{\beta} R$, $u' : Q \xrightarrow{\beta} S$, $t' : R \xrightarrow{\alpha} S$, *and* $t \;\iota\; u$, *then* $t \sim t'$.

We are now able to show independence of diamonds (ID), which can be seen as dual of SP.

**Definition 4.6 (Independence of Diamonds (ID)).** *An LTSI satisfies the Independence of Diamonds property (ID) if whenever we have a diamond* $t : P \xrightarrow{\alpha} Q$, $u : P \xrightarrow{\beta} R$, $u' : Q \xrightarrow{\beta} S$ *and* $t' : R \xrightarrow{\alpha} S$, *with*

- $Q \neq R$ *if* $\alpha$ *and* $\beta$ *are both forwards or both backwards;*
- $P \neq S$ *otherwise;*

*then* $t \;\iota\; u$.

**Proposition 4.7.** *If an LTSI satisfies BTI and CPI then it satisfies ID.*

We now consider the interaction between events and causal equivalence. We need some notation first.

**Definition 4.8.** *Let* $r$ *be a path in an LTSI* $\mathcal{L}$ *and let* $e$ *be an event of* $\mathcal{L}$. *Let* $\sharp(r, e)$ *be the number of occurrences of transitions* $t$ *in* $r$ *such that* $t \in e$, *minus the number of occurrences of transitions* $t$ *in* $r$ *such that* $t \in \underline{e}$.

We now show that $\sharp(r, e)$ is invariant under causal equivalent traces.

**Lemma 4.9.** *Let* $\mathcal{L}$ *be a pre-reversible LTSI. Let* $r \approx s$. *Then for each event* $e$ *we have that* $\sharp(r, e) = \sharp(s, e)$.

Lemma 4.9 generalises what was shown for the forward-only setting in [33, Corollary 4.3].

**Proposition 4.10.** *If an LTSI is pre-reversible, then for any rooted path* $r$ *and any forward event* $e$ *we have* $\sharp(r, e) \geq 0$.

## 4.2 CS and CL via Independence of Transitions

We first define causal safety and liveness using the independence relation.

**Definition 4.11.** *Let* $\mathcal{L} = (\mathsf{Proc}, \mathsf{Lab}, \rightarrow, \iota)$ *be a pre-reversible LTSI.*

1. *We say that* $\mathcal{L}$ *is* causally safe (CS) *if whenever* $P \xrightarrow{a} Q$, $r : Q \xrightarrow{\rho}_* R$, $\sharp(r, [P, a, Q]) = 0$ *and* $S \xrightarrow{a} R$ *with* $(P, a, Q) \sim (S, a, R)$, *then* $(P, a, Q) \;\iota\; t$ *for all* $t$ *in* $r$ *such that* $\sharp(r, [t]) > 0$.
2. *We say that* $\mathcal{L}$ *is* causally live (CL) *if whenever* $P \xrightarrow{a} Q$, $r : Q \xrightarrow{\rho}_* R$ *and* $\sharp(r, [P, a, Q]) = 0$ *and* $(P, a, Q) \;\iota\; t$, *for all* $t$ *in* $r$ *such that* $\sharp(r, [t]) > 0$, *then we have* $S \xrightarrow{a} R$ *with* $(P, a, Q) \sim (S, a, R)$.

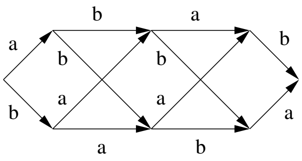

**Fig. 1.**

We may wish to close the independence relation over this axiom:

**Definition 4.12 (Independence Respects Events (IRE)).** *Whenever* $t \sim t' \iota u$ *we have* $t \iota u$*.*

IRE is one of the conditions in the definition of transition systems with independence [33, Definition 3.7]. Together with the axioms for pre-reversibility, it is enough to show both causal safety and causal liveness.

**Theorem 4.13.** *Let a pre-reversible LTSI satisfy IRE. Then it satisfies CS.*

**Theorem 4.14.** *Let a pre-reversible LTSI satisfy IRE. Then it satisfies CL.*

CS and CL are not derivable from CC; we give an example LTSI which satisfies CC but not CS and not CL.

*Example 4.15.* Consider the LTS in Figure 1. Independence is mostly coinitial and given by closing under BTI and CPI. Additionally we make the leftmost $a$-transition independent with all $b$-transitions. Note that all $a$-transitions belong to the same event, and all $b$-transitions belong to the same event. Also SP and WF hold, so that the LTSI is pre-reversible, and CC holds. However IRE does not hold. Furthermore CS fails using Definition 4.11. Indeed, consider any path $\overset{ba\underline{b}}{\rightarrow}_*$ from the start. CS would imply that the first $b$ is independent with the $a$ but this is not the case (we do have $\underline{b} \iota a$).

Also CL fails using Definition 4.11. Indeed, consider any path $\overset{abb}{\rightarrow}_*$ from the start. Since the leftmost $a$-transition is independent with all $b$-transitions, we should be able to reverse $a$ at the end of the path, but this is not possible.

The next axiom states that independence is fully determined by its restriction to coinitial transitions. This is related to axiom (E) of [33, page 325], but here we allow reverse as well as forward transitions.

**Definition 4.16 (Independence of Events is Coinitial (IEC)).** *If* $t_1 \iota t_2$ *then there are* $t'_1 \sim t_1$*,* $t'_2 \sim t_2$ *such that* $t'_1$ *and* $t'_2$ *are coinitial and* $t'_1 \iota t'_2$*.*

Thanks to previous axioms, independence behaves well w.r.t. reversing.

**Definition 4.17 (Reversing Preserves Independence (RPI)).** *If* $t \iota t'$ *then* $\underline{t} \iota t'$*.*

**Proposition 4.18.** *If an LTSI satisfies SP, CPI, IRE, IEC then it also satisfies RPI.*

All the axioms that we have introduced are independent, i.e. none is derivable from the remaining axioms.

**Proposition 4.19.** *SP, BTI, WF, CPI, IRE, IEC are independent of each other.*

### 4.3 CS and CL via Ordering of Events

To define CS and CL via ordering of events, we define the causality relation $\leq$ on events.

**Definition 4.20.** *Let* $\mathcal{L} = (\mathsf{Proc}, \mathsf{Lab}, \rightarrow, \iota)$ *be an LTSI. Let* $e, e'$ *be events of* $\mathcal{L}$*. Let* $e \leq e'$ *iff for all rooted paths* $r$*, if* $\sharp(r, e') > 0$ *then* $\sharp(r, e) > 0$*. As usual* $e < e'$ *means* $e \leq e'$ *and* $e \neq e'$*. If* $e < e'$ *we say that* $e$ *is a* cause *of* $e'$*.*

**Lemma 4.21.** *If an LTSI satisfies SP, BTI, WF and CPI then* $\leq$ *is a partial ordering on events.*

Previously, orderings on events have been defined using forward-only rooted paths; in fact, the definitions coincide for pre-reversible LTSIs.

**Definition 4.22 ([12,28]).** *Let* $\mathcal{L} = (\mathsf{Proc}, \mathsf{Lab}, \rightarrow, \iota)$ *be an LTSI. Let* $e, e'$ *be events of* $\mathcal{L}$*. Let* $e \leq_{\mathsf{f}} e'$ *iff for all rooted forward-only paths* $r$*, if* $r$ *contains a representative of* $e'$ *then* $r$ *also contains a representative of* $e$*.*

**Lemma 4.23.** *For any LTSI,* $e \leq e'$ *implies* $e \leq_{\mathsf{f}} e'$*. If an LTSI satisfies SP, BTI, WF and CPI then* $e \leq_{\mathsf{f}} e'$ *implies* $e \leq e'$*.*

*Proof.* Straightforward using PL and Lemma 4.9. □

We now give definitions of causal safety and causal liveness using ordering on events.

**Definition 4.24.** *Let* $\mathcal{L} = (\mathsf{Proc}, \mathsf{Lab}, \rightarrow, \iota)$ *be an LTSI.*

1. *We say that* $\mathcal{L}$ *is* ordered causally safe ($\mathrm{CS}_<$) *if whenever* $P \xrightarrow{a} Q$*,* $r : Q \xrightarrow{\rho}_* R$*,* $\sharp(r, [P, a, Q]) = 0$ *and* $S \xrightarrow{a} R$ *with* $(P, a, Q) \sim (S, a, R)$*, then* $[P, a, Q] \not< e'$ *for all* $e'$ *such that* $\sharp(r, e') > 0$*.*
2. *We say that* $\mathcal{L}$ *is* ordered causally live ($\mathrm{CL}_<$) *if whenever* $P \xrightarrow{a} Q$*,* $r : Q \xrightarrow{\rho}_* R$ *and* $\sharp(r, [P, a, Q]) = 0$ *and* $[P, a, Q] \not< e'$ *for all* $e'$ *such that* $\sharp(r, e') > 0$ *then we have* $S \xrightarrow{a} R$ *with* $(P, a, Q) \sim (S, a, R)$*.*

We postpone giving proofs of $\mathrm{CS}_<$ and $\mathrm{CL}_<$ until we have introduced a further definition of causal safety and liveness using independence of events.

### 4.4 CS and CL via Independent Events

We now introduce a third version of causal safety and liveness, which uses independence like CS and CL, but on events rather than on transitions. First we lift independence from transitions to events.

**Definition 4.25 (Coinitially independent events).** *Let events* $e, e'$ *be* (coinitially) independent, *written* $e \mathsf{ci} e'$, *iff there are coinitial transitions* $t, t'$ *such that* $[t] = e$, $[t'] = e'$ *and* $t \iota t'$.

**Lemma 4.26.** *If an LTSI is pre-reversible, then if* $e \mathsf{ci} e'$ *we have also* $\underline{e} \mathsf{ci} e'$.

Thus in pre-reversible LTSIs, $\mathsf{ci}$ is fully determined just considering forward events. By Lemma 4.26, if we know $e \mathsf{ci} e'$ then we know $\mathsf{und}(e) \mathsf{ci} \mathsf{und}(e')$.

We can give a third formulation of causal safety and liveness using $\mathsf{ci}$:

**Definition 4.27.** *Let* $\mathcal{L} = (\mathsf{Proc}, \mathsf{Lab}, \rightarrow, \iota)$ *be a pre-reversible LTSI.*

1. *We say that* $\mathcal{L}$ *is* coinitially causally safe *(*$CS_{\mathsf{ci}}$*) if whenever* $P \xrightarrow{a} Q$, $r : Q \xrightarrow{\rho}_* R$, $\sharp(r, [P, a, Q]) = 0$ *and* $S \xrightarrow{a} R$ *with* $(P, a, Q) \sim (S, a, R)$, *then* $[P, a, Q] \mathsf{ci} e$ *for all forward events* $e$ *such that* $\sharp(r, e) > 0$.
2. *We say that* $\mathcal{L}$ *is* coinitially causally live *(*$CL_{\mathsf{ci}}$*) if whenever* $P \xrightarrow{a} Q$, $r : Q \xrightarrow{\rho}_* R$ *and* $\sharp(r, [P, a, Q]) = 0$ *and* $[P, a, Q] \mathsf{ci} e$, *for all forward events* $e$ *such that* $\sharp(r, e) > 0$, *then we have* $S \xrightarrow{a} R$ *with* $(P, a, Q) \sim (S, a, R)$.

Note that in Definition 4.27 we operate at the level of events, rather than at the level of transitions as in Definition 4.11.

**Theorem 4.28.** *If an LTSI is pre-reversible then it satisfies* $CS_{\mathsf{ci}}$.

We now introduce a weaker version of axiom IRE (Definition 4.12).

**Definition 4.29 (Coinitial IRE (CIRE)).** *If* $[t] \mathsf{ci} [u]$ *and* $t, u$ *are coinitial then* $t \iota u$.

**Theorem 4.30.** *If a pre-reversible LTSI satisfies CIRE then it satisfies* $CL_{\mathsf{ci}}$.

We next give an example where CC holds but not $\mathrm{CS}_{\mathsf{ci}}$ (and not CPI).

*Example 4.31.* Consider the cube with transitions $a, b, c$ on the left in Figure 2, where the forward direction is from left to right. We add independence as given by BTI. So SP, BTI, WF hold, but not CPI. From the start we have an $a$-transition followed by a path $r = bc$ followed by $\underline{a}$. For $\mathrm{CS}_{\mathsf{ci}}$ to hold, we want $\underline{a}$ to be the reverse of the same event as the first $a$. They are connected by a ladder with sides $cb$. We add independence for all corners on the two faces of the ladder ($ab$ and $ac$). Then we get $bc \approx cb$ (independence at a single corner is enough). However the $b$s are not the same event since the $bc$ face does not have independence at each corner. Therefore we do not get $[a] \mathsf{ci} [b]$, and $\mathrm{CS}_{\mathsf{ci}}$ fails.

We next give an example where $\mathrm{CS}_{\mathsf{ci}}$ and $\mathrm{CL}_{\mathsf{ci}}$ hold but not CC.

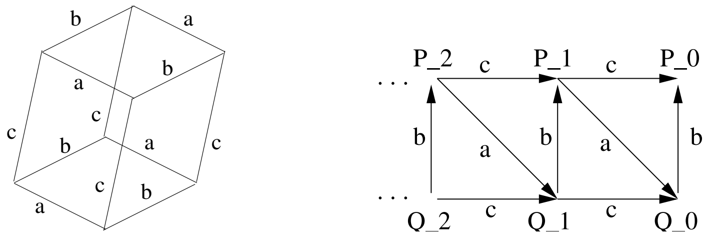

**Fig. 2.**

*Example 4.32.* Consider the LTSI with $Q_i \xrightarrow{b} P_i$, $P_{i+1} \xrightarrow{c} P_i$, $Q_{i+1} \xrightarrow{c} Q_i$, $P_{i+1} \xrightarrow{a} Q_i$ for $i = 0, 1, \ldots$. This is shown on the right in Figure 2. Clearly WF does not hold. We add coinitial independence to make BTI and CPI hold. Then also SP and CIRE hold. However, CC fails since, for example $P_1 \xrightarrow{a} Q_0 \xrightarrow{b} P_0$ and $P_1 \xrightarrow{c} P_0$ are coinitial and cofinal but not causally equivalent. Note that there are just three events $a, b, c$ with $a \mathrel{\mathsf{ci}} c$, $b \mathrel{\mathsf{ci}} c$ but not $a \mathrel{\mathsf{ci}} b$. $\mathrm{CS}_{\mathsf{ci}}$ and $\mathrm{CL}_{\mathsf{ci}}$ hold. Indeed, $c$ is independent from every other action, and it can always be undone, while $a$ and $b$ are independent from $c$ only and they can be undone after any path composed by $c$ and no others.

### 4.5 Polychotomy

In this section we relate our three versions of causal safety and liveness, with the help of what we call *polychotomy*, which states that if events do not cause each other and are not in conflict, then they must be independent. We start by defining a conflict relation on events.

**Definition 4.33.** *Two forward events $e, e'$ are in* conflict*, written $e \# e'$, if there is no rooted path $r$ such that $\sharp(r, e) > 0$ and $\sharp(r, e') > 0$.*

Much as for orderings, conflict on events has been defined previously using forward-only rooted paths [12,28]; in fact, the definitions coincide for pre-reversible LTSIs. We omit the details.

**Definition 4.34 (Polychotomy).** *Let $\mathcal{L}$ be a pre-reversible LTSI. We say that $\mathcal{L}$ satisfies* polychotomy *if whenever $e, e'$ are* forward *events, then exactly one of the following holds: 1. $e = e'$; 2. $e < e'$; 3. $e' < e$; 4. $e \# e'$; or 5. $e \mathrel{\mathsf{ci}} e'$.*

Property NRE below is related to polychotomy.

**Definition 4.35 (No Repeated Events (NRE)).** *In any rooted path $r$, for any forward event $e$ we have $\sharp(r, e) \leq 1$.*

**Lemma 4.36 (Polychotomy).** *Suppose that a pre-reversible LTSI satisfies NRE. Then polychotomy holds.*

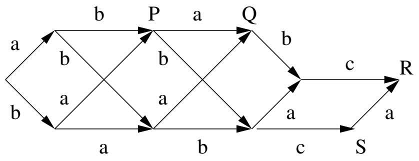

**Fig. 3.**

*Example 4.37.* Consider the LTSI in Figure 3. We add independence to make BTI and CPI hold. Both SP and WF hold. Hence, CC holds as well. There are three events, labelled with $a, b, c$. Clearly NRE fails for both $a$ and $b$. We see that $a < c$ but also $a \text{ ci } c$, so that polychotomy fails. $\text{CS}_{\text{ci}}$ holds by Theorem 4.28. However $\text{CS}_<$ fails: consider the transition $P \xrightarrow{a} Q$ together with the path $r : Q \xrightarrow{bc}_* R$ and $S \xrightarrow{a} R$, and note that $a < c$.

The next lemma allows us to connect ordered safety and liveness with coinitial safety and liveness.

**Lemma 4.38.** *Suppose that a pre-reversible LTSI satisfies NRE. Suppose $P \xrightarrow{a} Q$, $e = [P, a, Q]$, $r : Q \xrightarrow{\rho}_* R$ and $\sharp(r, e') > 0$ where $e'$ is a forward event. Then exactly one of $e \text{ ci } e'$ and $e < e'$ holds.*

**Proposition 4.39.** *Suppose that a pre-reversible LTSI $\mathcal{L}$ satisfies NRE. Then*

1. *$\mathcal{L}$ satisfies $CS_<$.*
2. *$\mathcal{L}$ satisfies $CL_{\text{ci}}$ iff $\mathcal{L}$ satisfies $CL_<$.*

Property RED below is also related to NRE and polychotomy.

**Definition 4.40.** *An LTSI satisfies* ***Reverse Event Determinism (RED)*** *if whenever $t, t'$ are backward coinitial transitions and $t \sim t'$ then $t = t'$.*

**Proposition 4.41.** *If a LTSI $\mathcal{L}$ is pre-reversible then the following are equivalent: 1. $\mathcal{L}$ satisfies NRE; 2. $\mathcal{L}$ satisfies RED; 3. independence $\text{ci}$ is irreflexive on events; and 4. polychotomy holds.*

**Proposition 4.42.** *Suppose that a pre-reversible LTSI satisfies CIRE. Then it also satisfies NRE.*

NRE was shown in the forward-only setting of occurrence transition systems with independence in [33, Corollary 4.6]. It was also shown in the reversible setting without independence in [28, Proposition 2.10].

*Example 4.43.* Consider the LTSI in Figure 4. Independence is given by closing under BTI and CPI. There are three events, labelled $a, b, c$, which are all independent of each other. We see that NRE holds but not CIRE. Also $\text{CL}_{\text{ci}}$ and $\text{CL}_<$ fail: consider $P \xrightarrow{a} Q \xrightarrow{b} R$, where $a$ cannot be reversed at $R$.

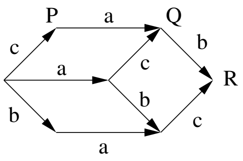

**Fig. 4.**

**Proposition 4.44.** *Let $\mathcal{L}$ be a pre-reversible LTSI.*

1. *If IEC holds then $CL_{\mathsf{ci}}$ implies CL.*
2. *If IEC and NRE hold then $CL_{<}$ implies CL.*

## 5 Coinitial Independence

In this section we consider coinitial LTSIs, defined as follows, and their relationship with LTSIs in general.

**Definition 5.1.** *Let $\mathcal{L} = (\mathsf{Proc}, \mathsf{Lab}, \rightarrow, \iota)$ be a combined LTSI. Then $\iota$ is* coinitial *if for all transitions $t, u$, if $t\ \iota\ u$ then $t$ and $u$ are coinitial. We say that $\mathcal{L}$ is coinitial if $\iota$ is coinitial.*

We define a mapping $c$ restricting general independence to coinitial transitions and a mapping $g$ extending independence along events.

**Definition 5.2.** *Given an LTSI $(\mathsf{Proc}, \mathsf{Lab}, \rightarrow, \iota)$, define $t\ g(\iota)\ u$ iff $t \sim t'\ \iota\ u' \sim u$ for some $t', u'$. Furthermore, define $t\ c(\iota)\ u$ iff $t\ \iota\ u$ and $t, u$ are coinitial.*

**Proposition 5.3.** *Let $\mathcal{L} = (\mathsf{Proc}, \mathsf{Lab}, \rightarrow, \iota)$ be a pre-reversible LTSI.*

1. *If $\mathcal{L}$ is coinitial and satisfies CIRE then $\mathcal{L}' = (\mathsf{Proc}, \mathsf{Lab}, \rightarrow, g(\iota))$ is a pre-reversible LTSI and satisfies IRE and IEC.*
2. *if $\mathcal{L}$ satisfies IRE then $\mathcal{L}' = (\mathsf{Proc}, \mathsf{Lab}, \rightarrow, c(\iota))$ is a pre-reversible coinitial LTSI and satisfies CIRE.*

Thanks to Proposition 5.3, we can extend a coinitial pre-reversible LTSI satisfying CIRE in a canonical way to a pre-reversible LTSI satisfying IRE and IEC.

In some reversible calculi (such as RCCS) independence of coinitial transitions is defined purely by reference to the labels. If this is the case it is a simple matter to verify the axioms CPI and CIRE.

**Proposition 5.4.** *Let $\mathcal{L} = (\mathsf{Proc}, \mathsf{Lab}, \rightarrow, \iota)$ be a coinitial combined LTSI. Suppose that $I$ is a binary relation on* Lab *such that for any coinitial transitions $t : P \xrightarrow{\alpha} Q$ and $u : P \xrightarrow{\beta} R$ we have $t\ \iota\ u$ iff $I(a, b)$, where $a$ and $b$ are the underlying labels $a = \mathsf{und}(\alpha)$, $b = \mathsf{und}(\beta)$. Then $\mathcal{L}$ satisfies CPI and CIRE.*

*Proof.* Straightforward, noting that labels on opposite sides of a diamond of transitions must be equal. □

Note that $I$ must be irreflexive, since $\iota$ is irreflexive.

If we have a coinitial pre-reversible LTSI satisfying CIRE then $\mathrm{CS}_<$ and $\mathrm{CL}_<$ hold (using Proposition 4.42 and Proposition 4.39). Applying mapping $g$ we get a general pre-reversible LTSI satisfying IRE and IEC by Proposition 5.3. This will satisfy CS and CL as a result of applying Theorem 4.13 and Theorem 4.14 respectively. It will also satisfy $\mathrm{CS}_<$ and $\mathrm{CL}_<$. Conversely, if we have a general pre-reversible LTSI satisfying IRE then CS and CL hold by Theorem 4.13 and Theorem 4.14 respectively. Applying mapping $c$ we get a coinitial pre-reversible LTSI satisfying CIRE. This will satisfy $\mathrm{CS}_<$ and $\mathrm{CL}_<$ .

## 6 Case Studies

We look at whether our axioms hold in various reversible formalisms. Remarkably, all the works below provide proofs of the Loop Lemma.

**RCCS** We consider here the semantics of RCCS in [6], and restrict the attention to coherent processes [6, Definition 2]. In RCCS, transitions $P \stackrel{\mu:\zeta}{\to} Q$ and $P \stackrel{\mu':\zeta'}{\to} Q'$ are concurrent if $\mu \cap \mu' = \emptyset$ [6, Definition 7]. This allows us to define coinitial independence as $t \;\iota\; u$ iff $t$ and $u$ are concurrent. We now argue that the resulting coinitial LTSI is pre-reversible and also satisfies CIRE. SP was shown in [6, Lemma 8]. BTI was shown in the proof of [6, Lemma 10]. WF is straightforward, noting that backward transitions decrease memory size. Hence, we obtain a very much simplified proof of CC. For CPI and CIRE we note that independence is defined on the underlying labels and thus Proposition 5.4 applies. Therefore $\mathrm{CS}_<$ and $\mathrm{CL}_<$ hold. Using Proposition 5.3, we can get an LTSI with general independence satisfying IRE and IEC, and therefore CS and CL. This is the first time these causal properties have been proved for RCCS.

**HO$\pi$** We consider here the uncontrolled reversible semantics for HO$\pi$ [18]. We restrict our attention to reachable processes, called there consistent. The semantics is a reduction semantics; hence there are no labels (or, equivalently, all the labels coincide). To have more informative labels we can consider the transitions defined in [18, Section 3.1], where labels are composed of memory information and a flag denoting whether the transition is forward or backward. The notion of independence would be given by the concurrency relation on coinitial transitions [18, Definition 9]. All pre-reversible LTSI axioms hold, as well as CIRE which is needed for causal safety and liveness. Specifically, SP is proved in [18, Lemma 9]. BTI holds since distinct memories have disjoint sets of keys [18, Definition 3 and Lemma 3] and by the definition of concurrency [18, Definition 9]. WF holds as each backward step consumes a memory, which is finite to start with. Finally, CPI and CIRE are valid since the notion of concurrency is defined on the annotated labels and using our Proposition 5.4.

As a result we obtain a very much simplified proof of CC. Moreover, using CPI and CIRE, we get the $CS_<$ and $CL_<$ safety and liveness properties and, applying mapping $g$ from Section 5, we get a general pre-reversible LTSI satisfying IRE and IEC, hence CS and CL are satisfied. This is the first time that causal properties have been shown for HO$\pi$.

**R$\boldsymbol{\pi}$** We consider the (uncontrolled) reversible semantics for $\pi$-calculus defined in [5]. We restrict the attention to reachable processes. The semantics is an LTS semantics. Independence is given as concurrency which is defined for consecutive transitions [5, Definition 4.1]. CC holds [5, Theorem 4.5].

Our results are not directly applicable to R$\pi$, since SP holds up to label equivalence of transitions on opposite sides of the diamond, rather than equality of labels as in our approach. We would need to extend axiom SP and the definition of causal equivalence to allow for label equivalence in order to handle R$\pi$ using our axiomatic method.

**Erlang** We consider the uncontrolled reversible (reduction) semantics for Erlang in [20]. We restrict our attention to reachable processes. In order to have more informative labels we can consider the annotations defined in [20, Section 4.1]. We then can define coinitial transitions to be independent if they are concurrent [20, Definition 12].

We next discuss the validity of our axioms in reversible Erlang. SP is proved in [20, Lemma 13] and BTI is trivial from the definition of concurrency [20, Definition 12]. WF holds since the pairs of integers (total number of elements in memories, total number of messages queued) ordered under lexicographic order are always positive and decrease at each backward step. Intuitively, each step but the ones derived using the rule for reverse sched (see [20, Figure 11]) consumes an item of memory, and each step derived using rule reverse sched removes a message from a process queue. Finally, CPI and CIRE hold since the notion of concurrency is defined on the annotated labels, and by Proposition 5.4.

Since this the setting is very similar to the one of HO$\pi$ (both calculi have a reduction semantics and a coinitial notion of independence defined on enriched labels), we get the same results as for HO$\pi$, including CC, and CS and CL.

**Reversible occurrence nets** Reversible occurrence nets [25,24] are traditional occurrence nets (safe and with no backward conflicts) extended with a reverse transition for each forward transition. They give rise to an LTS where states are pairis $(N, m)$ with $N$ a net and $m$ a marking. A computation that represents firing a transition $t$ in $(N, m)$ and resulting in $(N, m')$ is given by a firing relation $(N, m) \xrightarrow{t} (N, m')$. The notion of independence is the concurrency relation [25, Section 3] which is defined between arbitrary firings (transitions). Hence, we get a general LTSI. The CC property is shown by following the traditional approach in [6]. SP and PL are shown as well. PL and CC require several pages of proofs [24]. The causal safety and causal liveness properties are not considered in [25,24].

We can obtain CC, and additionally CS and CL, as follows. SP and BTI are proved for reversible occurrence nets in [24] as Lemma 4.3 and Lemma 3.3

respectively. WF holds because there are no forward cycles of firings in occurrence nets, hence no infinite reverse paths. In order to have CS and CL, we need to show CPI and IRE. Lemma 3.4 in [24] gives CPI. Events can be defined on firings as in our Definition 4.5, and then IRE holds as the concurrency relation preserves such events.

## 7 Conclusion, Related and Future Work

The literature on causal-consistent reversibility (see, for example the early survey [19]) has a number of proofs of results such as the parabolic lemma (PL) and the causal consistency property (CC), all of which are instantiated to a specific calculus, language or formalism. We have taken here a complementary approach, analysing the properties of interest in an abstract and language-independent setting. In particular, we have shown how to prove the most relevant of these properties from a small number of axioms.

Our approach builds upon [28], where a set of axioms for reverse LTSs was given and several interesting properties were shown. While the idea is similar, the development is rather different since we consider more basic axioms (we only share WF, while many of the axioms in [28], such as UT, follow from ours), and since the two papers focus on different properties. We focus on CC and various forms of CS and CL, while [28] considers correspondence with prime event structures and reversible bisimulations. Moreover, LTSs in [28] do not have a notion of independence.

In other related work, we may particularly mention [8], which like ours takes an abstract view, though based on category theory. However, its results concern irreversible actions, and do not provide insights in our setting, where all actions are reversible. The only other work which takes a general perspective is [3], which concentrates on how to derive a reversible extension of a given formalism. However, proofs concern a limited number of properties (essentially our CC), and hold only for extensions built using the technique proposed there. Also [27,29] are general, since they propose how to reverse a calculus that can be defined in a general format of SOS rules. However, the format has its syntactic constraints while our approach abstracts from them. Finally, [9] presents a number of properties such as, for example, backward confluence, which arise in the context of reversing of steps of executed transitions in Place/Transition nets.

The approach proposed in this paper opens a number of new possibilities. Firstly, when devising a new reversible formalism, our results provide a rich toolbox to prove (or disprove) relevant properties in a simple way. This is particularly relevant since causal-consistent reversibility is getting applied to more and more complex languages, such as Erlang [20], where direct proofs become cumbersome and error-prone. Secondly, our abstract proofs are relatively easy to formalise in a proof-assistant, which is even more relevant given that this will certify the correctness of the results for many possible instances. Another possible extension of our work concerns integrating into our framework irreversible actions [7]. In order to do that we could take inspiration from the above-mentioned [8].
